# Case of Acute Rheumatism—Pericarditis and Pneumonia

**Published:** 1869-05-01

**Authors:** Fred. B. Wood


### *Case of Acute Rheumatism—Pericarditis and Pneumonia.*

BY DR. FRED. B. WOOD.

BIG RAPIDS, MICHIGAN.

EDITORS JOURNAL:

I was called, Feb. 15th, to visit Mrs. T., age 24, American. The patient stated that her health had been good until about the 10th, when she was attacked with pain and tenderness in the left knee joint, afterwards in the right knee, right ankle and wrist they all being swollen and reddened at my first visit. A part of the time since her attack she had suffered from anorexia, thirst, and severe pain in the præcordia, for which a sinapism had been applied. Aside from this she had received no treatment. I immediately began the alkaline treatment with potash and soda, with small doses of colchicum.

Feb. 24.—On this date there was present a well-marked pericardial friction murmur, with the symptoms of acute pericarditis. The pulse was 124, respirations 60. There was a sharp pain in the præcordia on deep inspiration. In connection with the above treatment four ounces of whisky was directed daily, with sufficient opium to procure relief.

25.—Patient more comfortable. Pulse 120, respirations 54. The affected joints were all painful and tender. The friction murmur was louder than on the previous day. The treatment was continued.

26.—Pulse was 114, respiration 40. Treatment the same.

27.—No change.

28.—Suffered more pain in the præcordia, which was quite tender. Affected joints tender but not painful.

March 1.—There has been no alteration until this date, when she became much worse. The pulse 180; very feeble. She was greatly prostrated, and almost moribund; also complained of great pain in the chest.

The examination being extended revealed the signs of pneumonia in the lower lobe of the left lung. Marked dullness on percussion, and the bronchial respiration existed over this lobe. Impending dissolution denoted. The lips were livid, and the face had a dusky hue. I now withdrew all treatment excepting whisky, opium and ammonia.

2.—One p. m. Pulse 126, respiration 58. Resting well since nine a. m. Has taken half an ounce of whisky, five grains of carbonate of ammonia, and ten grains of chlorate of potassa every half hour.

3.—Some improvement. On this date I suspended the use of the chlorate of potassa and began giving sul. morphia, $\frac{1}{8}$ of a grain every hour; other treatment the same.

4.—Pulse 116, respiration 42. Complains of but little pain. Rests well.

5.—Complains of pain more severe. Bronchial breathing continued over lower lobe of left lung.

I now increased the whisky to one ounce each hour. Carbonate of ammonia 15 grains, morphia sul. $\frac{1}{4}$ grain, also to be given each hour.

6.—Improvement marked. Pulse 112, respirations 38. Complains of no pain; rests well. I ordered beef tea, eggs and milk in moderate quantities. From this date until the eleventh there was but little change, when the respirations had become vesicular over the whole of the affected lobe of the affected lung. Some soreness but no pain in the joints. The friction murmur has almost entirely disappeared.

Pulse 100, respirations 30. I ordered whisky ℥ viij to be taken each day. Eggs 4, milk oj, and beef tea o. ss. each day.

15.—Saw my patient to-day. She considers herself almost well. A well-regulated, full diet ordered. Patient dismissed.

Remarks.—I have given a condensed account of the history of this case from day to day, after the development of pericarditis and pneumonia, up to the time when the improvement was fairly under way, with reference to the treatment employed.

The treatment was determined by the existence of the affections just named, without regard to the fact that these affections were developed in the course of rheumatism.

How far the measures employed contributed to the favorable issue of the case I leave the reader to judge.

